# PKR modulates abnormal brain signaling in experimental obesity

**DOI:** 10.1371/journal.pone.0196983

**Published:** 2018-05-24

**Authors:** Mariko Taga, François Mouton-Liger, Malha Sadoune, Sarah Gourmaud, Jenny Norman, Marion Tible, Sylvie Thomasseau, Claire Paquet, James A. R. Nicoll, Delphine Boche, Jacques Hugon

**Affiliations:** 1 Clinical Neurosciences, Clinical and Experimental Sciences, Faculty of Medicine, University of Southampton, Southampton, United Kingdom; 2 INSERM Units U942, Paris, France; 3 Histochemistry Research Unit, Clinical and Experimental Sciences, Faculty of Medicine, University of Southampton, Southampton, United Kingdom; 4 Center of Cognitive Neurology Lariboisière Hospital, APHP, University Paris Diderot, Paris, France; 5 Department of Cellular Pathology, University Hospital Southampton NHS Foundation Trust, Southampton, Southampton, United Kingdom; Universidade do Estado do Rio de Janeiro, BRAZIL

## Abstract

Metabolic disorders including obesity and type 2 diabetes are known to be associated with chronic inflammation and are obvious risk factors for Alzheimer’s disease. Recent evidences concerning obesity and diabetes suggest that the metabolic inflammasome (“metaflammasome”) mediates chronic inflammation. The double-stranded RNA-dependent protein kinase (PKR) is a central component of the metaflammasome. In wild type (WT) and PKR^-/-^ mice, blood glucose, insulin and lipid levels and the brain expression of the phosphorylated components of the metaflammasome—PKR, JNK, IRS1 and IKKbeta—were studied after the induction of obesity by a high fat diet (HFD). The results showed significant increased levels of activated brain metaflammasome proteins in exposed WT mice but the changes were not significant in PKR^-/-^ mice. In addition, gain weight was observed in WT mice and also in PKR^-/-^ mice exposed to HFD. Increased blood insulin level was more accentuated in PKR ^-/-^ mice. The modulation of PKR activity could be an appropriate therapeutic approach, aimed at reducing abnormal brain metabolism and inflammation linked to metabolic disorders in order to reduce the risk of neurodegeneration.

## Introduction

Metabolic diseases (e.g. obesity, type 2 diabetes and dyslipidemia) and dementia are currently amongst the largest public health problems in industrialized countries. Type 2 diabetes is characterized by peripheral insulin resistance leading to hyperinsulinemia with disrupted glucose homeostasis and is associated with a chronic inflammation [1]. The link between metabolic disorders and inflammation was described by Hotamisligil and his colleagues by the term metaflammation [2], which was proposed to be a low-grade inflammation orchestrated by metabolic cells and induced by endoplasmic reticulum stress, lipid stress (e.g. obesity, type 2 diabetes, dyslipidemia) or infectious stress. A group of proteins was identified as major players linking nutrient stress, inflammation and metabolic regulation and was called metaflammasome [3]. According to this study, the protein PKR (double stranded RNA binding protein kinase) is a key component of the metaflammasome and plays an important role in the regulation of the others components of the metaflammasome including JNK (c-Jun N-terminal kinase), IRS1 (Insulin receptor substrate 1) and IKKbeta (IκB kinase beta). The pathological activation of metaflammasome proteins was reported in peripheral organs of obese mice, but not in PKR knock-out mice demonstrating the impairment of metaflammasome activity in the context of metabolic disorders [3] [4]. Recently this finding has been debated in a new study showing the absence of PKR control on experimental obesity and associated abnormal metabolisms [5]. In addition to the role of PKR in metabolic regulation and inflammatory response, this protein is also known to play a central role in cellular apoptosis and in the control of viral infection by phosphorylating eIF2alpha (Eukaryotic Initiation Factor 2 alpha) leading to the inhibition of protein synthesis [6, 7].

In the last decade, the number of studies describing the relationship between brain lesions and metabolic disorders has increased, highlighting the fact that metabolic disorders such as obesity and type 2 diabetes could be risk factors for the development of Alzheimer’s Disease (AD) for example [8–11]. Previous studies have observed that the risk of late-onset AD was 65% higher in people with type 2 diabetes than in non-diabetic people [12] and that the rate of cognitive decline was accelerated in elderly people with type 2 diabetes [13]. However, the molecular mechanisms at the origin of this association are still unclear. AD shares some pathophysiological features with type 2 diabetes including insulin resistance and disruption of glucose metabolism [14, 15]. Interestingly, several studies support a role for PKR in AD patients and in experimental models. Its activated form was found increased (i) in neural cell models exposed to Abeta42 [7], (ii) in APP_SL_/presenilin 1 knock-in mice [16], and (iii) in the brain and cerebrospinal fluid (CSF) of AD patients [17] [16]. A link between CSF activated PKR levels and cognitive decline was recently highlighted [18]. The putative role of activated JNK3 in AD was also revealed by increased JNK3 concentrations in the brain and CSF of AD patients [19]. Therefore, according to these findings, metaflammasome proteins might play a detrimental function in AD brain similarly to the one described in peripheral organs during metabolic disorders. We have shown recently that metaflammasome proteins were abnormally expressed in human AD brains [20]. Therefore, according to these findings, metaflammasome proteins might play a detrimental function in AD brains similarly to the one described in peripheral organs during metabolic disorders.

Consequently, the goal of this study was to assess the levels of brain metaflammasome proteins in experimental metabolic disorders linked to experimental obesity and to determine if PKR controls this process using PKR knockout (PKR^-/-^) mice. Our results suggest that PKR can modulate the cerebral expression of metaflammasome proteins in obese mice.

## Materials and methods

### Animals

In this study, the animal experimentations were conducted in agreement with accepted standards of animal care, as outlined in the NIH Guide for the Care and Use of Laboratory Animals. Experiments were approved by the Ethics Committee (Ethics Committee for Animal Experimentation Lariboisiere-Villemin of the Faculty of Medicine Paris 7, and Inserm France Paris, France).

PKR^-/-^ mice developed normally, were fertile and no difference with wild type mice were noted in term of behavior and phenotypes. Histological examination of internal organs was normal. These mice have not an overt abnormal immunological phenotype profile [21].

Adult male wild-type (WT) C57BL/6J mice were purchased from Charles River Breeding (Charles River, France). The RNA-binding domain defective mice, PKR^-/-^ mice (C57Bl/6J background) were used to analyze the role of PKR [22]. On arrival, animals were housed by groups of 3–4 animals, with food and water *ad libitium*, in a room controlled with a 12:12hr light-dark cycle.

Three-week old male WT C57BL/6J and PKR^-/-^ mice were fed with a high fat diet *ad libitium* (protein 20kcal%; carbohydrate 20kcal%; Fat 60kcal % (cat: D12492)) for 16 weeks (at least n = 6 for each group). The wild type control group received a standard rodent diet *ad libitium* (protein 20kcal%; carbohydrate 70kcal%; lipid 10kcal % (cat: D12450B)) at the same time. Both diets were purchased from Research Diets (Research Diets, *Denmark*). Young mice were chosen in order to avoid modifications of brain metabolism that can occur with aging.

### Body weight assessment

Body weight was measured every week during the 16 weeks of the high fat diet experiment.

### Oral glucose tolerance test

In order to glucose status in mice, OGT was performed in control and PKR^-/-^ mice. After 16 hours of fasting, 5g/kg of glucose was orally administrated in control (n = 5). Blood glucose levels were assessed with an ACCU-Check Performa device (Roche Diagnostic Mannheim Germany) prior to oral administration and at 10, 20, 40, 90, 120, and 180 minutes after the oral feeding.

### Experimental procedures

3 hours after fasting, the blood glucose level was measured by puncture of the tail vein using an Accu-Check glucometer (One Touch; Roche Diagnostics, Meylan, *France*). Blood samples were collected in EDTA tubes and after deep anesthesia, animals were transcardially perfused with NaCl solution. Brains were rapidly removed from the animals. Half of the brain was dissected to isolate cortex and then placed in Eppendorf tubes, snap frozen on liquid nitrogen and stored at -80°C for western blot and TNFalpha detection. The other half-brain was stored in 4% PFA for immunohistochemistry.

#### Measurement of serum parameters

Blood samples, collected in EDTA tubes, were centrifuged (3000g, 20 min) just after collection. Serum levels of cholesterol, triglycerides, high-density lipoprotein (HDL) and low-density lipoprotein (LDL) were analyzed using an Architech c8000 autoanalyzer (Abbott, Rungis, France). Serum insulin was determined using an ELISA kit (CrystalChem, Downers Grove, IL, USA).

### Western blot

Brain lysates were prepared in RIPA buffer containing 10mM NaPi buffer, pH 7.8, 59mM NaCl, 1% Triton 100X, 0.5% DOC (Deoxycholic Acid), 0.1% of SDS (Sodium Dodecyl Sulfate), 10% of glycerol, 0.1μM calyculin (Serine/Threonine phosphatase inhibitor; *Cell Signaling*), 1mM sodium orthovanadate (Tyrosine phosphatase inhibitor; *Sigma*) and 1X protease inhibitor (*Roche Diagnostics GmbH*, *Penzberg*, *Germany*). The lysate was sonicated on ice 4 times for 5 seconds each on a medium setting and centrifuged at 16000g for 5 minutes at 4^°^C. The quantity of total protein was determined with a Micro BCA Protein Assay kit (*Thermo Scientific*, *Cergy-Pontoise*, *France*).

The protein samples (40 to 50 μg) were separated on 4–12% Mini-PROTEAN TGX precast gels *(Bio-Rad)* and transferred to nitrocellulose membranes *(GE Healthcare*, *Chalfont St*. *Giles*, *UK)*. Membranes were blocked for 1 hour in a Blocking Buffer (Casein Blocking Buffer 10X, *Sigma Aldrich*). After incubation with the primary antibody, membranes were incubated with secondary antibodies, sheep anti-mouse (1:5000, ECL Mouse IgG produced in sheep, HRP-Linked Whole Ab, *GE Helthcare*) or goat anti-rabbit (1:10000 Anti-Rabbit IgG) (whole molecule)- chemiluminescent signals. Membranes were then incubated in a solution of Amersham ECL Prime Western Blotting Detection Reagent (*GE Helathcare*) for 5 minutes. Bound proteins were visualized with Image Reader LAS 3000 (*Fujifilm*, *Courbevoie*, *France*).

### Immunohistochemistry

Four μm sections prepared from paraffin blocks were transferred to baths of clearene, then rehydrated though graded alcohol (100%-95%-70%) and immersed in water. Endogenous peroxidase activity was inhibited with incubation of 3% hydrogen peroxide in methanol. Heat-induced epitope retrieval (citrate or EDTA) was performed according to the protocol optimized for the primary antibody to be used. Sections were incubated overnight at 4^°^C or room temperature with the primary antibody. The following day, the appropriate secondary biotinylated antibody was applied followed by incubation with VECTASTAIN Elite ABC Kit (Vector Laboratories). DAB (3, 3-diaminobenzidine) (Vector Laboratories) was used to reveal specific staining.

### ELISA

The relations between TNKalpha and PKR have been previously explored [21, 23, 24]. The ELISA kit for mouse TNFalpha was purchased from Signosis (EA-2203; Euromedex, France). 100μL of each sample were dispatched in TNFalpha pre-coated wells and incubated for 1h at room temperature (RT). Three washes were performed; 100μL of biotinyled antibody were added and left for incubation for 1h at RT. Three washes were repeated. Streptavidin mix was then added to the wells and left for incubation for 45 min at RT. Three washes were finally performed and 100μL of substrate were added to the wells. Incubation for 20 min, in the dark at RT and 50μl of STOP solution were added. OD was determined at 450nm, directly after the addition of the STOP solution.

### Antibodies

The following antibodies were used:

#### Western blot

Anti-PKR (sc-708, *Santa Cruz*), anti-peIF2alpha (44728G, *Invitrogen*), anti-eIF2alpha (#9772, *Cell Signaling*), anti-pJNK (#4668, *Cell Signaling*), anti-JNK (sc-7345, *Santa Cruz*), anti-pIRS1 (07–247, *Millipore*), anti-IRS1 (sc-559, *Santa Cruz*), anti-pIKKbeta (#2694, *Cell Signaling*), anti-IKKbeta (#2370, *Cell Signaling*), and anti-Tubulin (T9026, *Sigma*).

#### Immunohistochemistry

Anti-pJNK (sc-6254, *Santa Cruz*), anti-pIRS1 (ab66154, *Abcam*), anti-pIKKbeta (OAEC00312), anti-Iba1 (019–19741, *Wako*).

### Quantification

#### Western blot

Membranes were quantified with multigauge software (Fujifilm). All data were normalized on tubulin. Results were evaluated with phosphorylated form to total form and presented as the ratio between the phosphorylated form to the full-length form.

#### Immunohistochemistry

10 images were captured at objective magnification x20 in the cerebral cortex of each animal. The software ImageJ, a Java-based image processing program developed at the National Institutes of Health was used to quantify the levels of immunostaining, which are presented as % protein load. The combined area occupied by all objects on the image, was recorded as a percentage (%) of the total area of the image and expressed as the percentage of protein load.

### Statistical analysis

Statistical analysis was performed using the statistical software GraphPad Prism (GraphPad Software, La Jolla California USA). The two-way ANOVA (with Tukey’s multiple comparison test) was used in order to study the effect of the strain of animal / or diet on the expression of a cerebral metaflammasome. To assess the effect of the HFD on the body weight, the two-way ANOVA repeated measure was performed (Newman-Keuls method). Results were considered significant when p<0.05. All experiments were done by researchers in a blind manner, not knowing the origin of the samples tested.

## Results

### Effect of HFD on body weight

**Fig 1:**
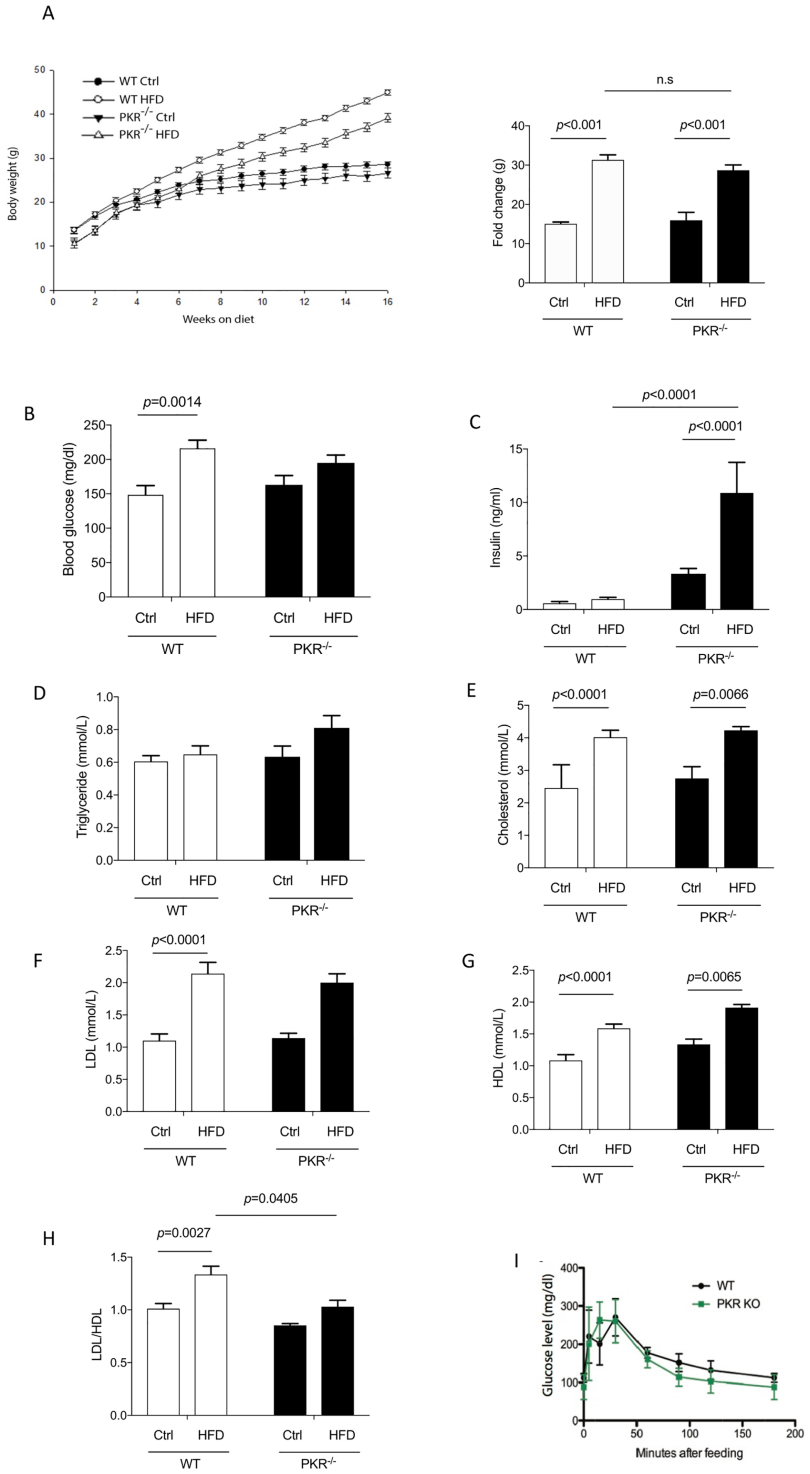
**(A)** Evolution of total body weight on control diet or HFD in WT and PKR^-/-^ mice and body weight gain during diet. Obesity is induced by HFD. Error bars = SEM. **(B)** Analysis of blood glucose level after 16 weeks of HFD in WT mice and PKR^-/-^ mice showing a significantly increased glucose level in WT mice after HFD treatment (*p* = 0.0014). Error bars = SEM. **(C)** Analysis of insulin level after 16 weeks of HFD in WT mice and PKR^-/-^ mice showing a significantly increased insulin level after HFD treatment in PKR ^-/-^ mice (*p<*0.0001). Error bars = SEM. **(D)** Analysis of triglyceride level after 16 weeks of HFD in WT mice and PKR^-/-^ mice shows no difference of triglyceride level in WT mice and PKR^-/-^ mice after HFD treatment. Error bars = SEM. **(E)** Analysis of cholesterol level after 16 weeks of HFD in WT mice and PKR^-/-^ mice showing a significant increased cholesterol level in WT HFD and PKR^-/-^ HFD compared to their controls. Error bars = SEM. **(F)** Analysis of LDL level after 16 weeks of HFD in WT mice and PKR^-/-^ mice showing a significant increased LDL level after HFD in WT mice compared to control diet (*p*<0.0001). Error bars = SEM. **(G)** Analysis of HDL level after 16 weeks of HFD in WT mice and PKR^-/-^ mice showing a significant increased HDL level after HFD in WT and PKR^-/-^ mice compared to controls. Error bars = SEM. **(H)** Analysis of ratio of LDL/HDL after 16 weeks of HFD in WT mice and PKR^-/-^ mice. The results showed a significant difference after HFD treatment in the WT mice (*p*<0.0027) but not in PKR^-/-^ mice, as well as between WT HFD and PKR^-/-^ HFD mice (*p* = 0.0405). Error bars = SEM. **(I)** The oral glucose tolerance test (glucose mg/dl) shows no significant difference between control mice and PKR knockout mice from T0 to T180 minutes. (n = 5).

Animals were fed with a HFD for 16 weeks, resulting an increase of body weight in WT and PKR^-/-^ mice. In WT mice, a significantly increased body weight was detected from the 5^th^ week (*p* = 0.017) and from 8^th^ weeks for PKR^-/-^ mice (*p* = 0.013). Furthermore, from the 5^th^ week onwards, the mean weight of PKR^-/-^ HFD mice was significantly lower than the mean weight of WT HFD mice (*p* = 0.004) and at the end of the study, the mean body weight of the PKR^-/-^ HFD mice was 17% lower than the one evaluated in WT HFD mice (*p*<0.001) (Fig 1A). But if we take into account the mean body weight at the beginning of the study, PKR K^-/-^ mice have a lower level (10,48g) than WT mice (13,63g). At the end of the HFD period PKR^-/-^ mice have gain 3.7 times of their original weight (39,17) and WT have gained 3.2 times (44,52g). There is no significant difference of weight between WT HFD and PKR^-/-^ HFD mice, suggesting that PKR does not control gain weight during HFD exposure in mice. No significant differences in body weight were observed between WT and PKR^-/-^ control diet mice (Fig 1A).

### Effect of PKR on HFD and metabolic regulation

#### Glucose and insulin levels

We observed that in WT mice, HFD led to a significant increase of blood glucose levels in WT HFD mice compared to WT control mice (*p* = 0.0014) (Fig 1B). This result confirms that WT mice fed a HFD developed type 2 diabetes. Interestingly, in PKR^-/-^ mice, a slight tendency to increase of blood glucose level but not reaching significant difference was observed between PKR^-/-^ HFD mice and PKR^-/-^ control diet mice (Fig 1B). According to this tendency finding, PKR seems to interfere perhaps partially with the modulation of blood glucose in HFD.

Surprisingly, HFD treatment did not induce a significant increase of insulin level (Fig 1C) in WT HFD mice compared to the WT Ctrl mice. However, the HFD treatment induced a significant increase of insulin levels in PKR^-/-^ mice, which is 8 times higher in the plasma of PKR^-/-^ mice fed with HFD compared to PKR^-/-^ mice with control diet (*p<*0.0001). In addition, the insulin level of PKR^-/-^ HFD mice was significantly higher than WT HFD mice (*p<*0.0001).

In order to determine whether PKR^-/-^ mice were insulin resistant, an oral glucose tolerance test was performed in WT and PKR^-/-^ mice. The results are depicted in Fig 1I and no significant difference was detected between both strains. The modifications of insulin secretion in PKR ^-/-^ mice are only detected after 16 weeks of HFD.

#### Cholesterol level

A significant increase of total cholesterol level was observed in WT HFD mice (*p*< 0.0001) and PKR^-/-^ HFD mice (*p* = 0.0066) (Fig 1E) suggesting that PKR might not have a role in total cholesterol level during lipid stress. In addition, the level of triglyceride concentrations displayed no variation after HFD treatment in both strains (Fig 1D), suggesting that HFD did not have an effect on the triglyceride concentration in our model. With the aim of studying HFD-induced dyslipidemia, the levels of HDL (High density lipoprotein) and LDL (Low density lipoprotein) were measured in the plasma of the mice. The mean LDL level was significantly increased in WT HFD mice (*p*<0.0001) mice but not in PKR^-/-^ mice, however no significant difference was observed between WT HFD mice and PKR^-/-^ HFD mice (Fig 1F). The analyses of HDL levels also showed a significant increase in WT HFD mice (*p*<0.0001) and in PKR^-/-^ HFD mice (*p* = 0.0065) (Fig 1G). Interestingly, the analysis of the ratio of LDL/HDL showed a significant increase of LDL/HDL ratios in WT HFD mice (*p* = 0.0027) but not in PKR^-/-^ HFD mice. In addition, the LDL/HDL ratios were significantly lower in PKR^-/-^ HFD mice compared to WT HFD mice (*p* = 0.0405) (Fig 1H). These data suggest that the knock-out of PKR has a beneficial effect on HDL metabolism in obese mice.

### Effect of HFD on microglia

**Fig 2:**
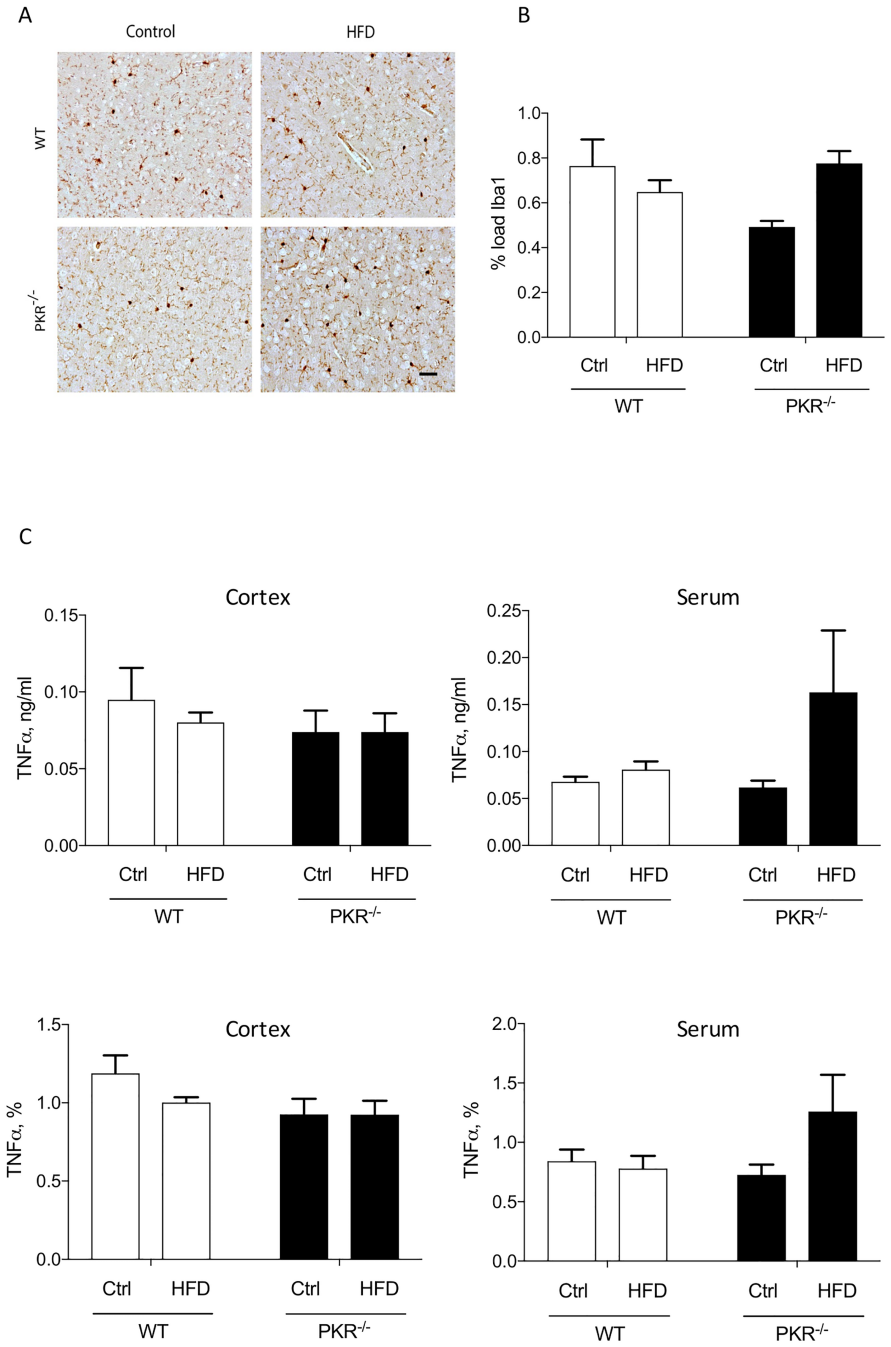
**(A)** Illustration of Iba1 immunostaining in the cortical grey matter in WT and PKR^-/-^ mice control or treated by HFD. Scale bar = 50μm. **(B)** Histogram of level of Iba1 protein load in the cortical grey matter of WT and PKR^-/-^ mice after 16 weeks of HFD. No significant modification of Iba1 expression is observed in both strains. Error bars = SEM. **(C)** Histogram of level of TNFalpha in the cortex and serum of WT and PKR^-/-^ mice after 16 weeks of HFD. (n = 5).

In both strains, no significant difference of Iba1 load (Fig 2A and 2B) after HFD was detected in the cortex. Furthermore, serum and brain TNFalpha levels were not significantly different among the four groups of mice (Fig 2C). These findings argue in favor of an absence of obvious abnormal detectable peripheral or brain inflammations with the used assessments in the four groups of mice.

### Effect of HFD on the expression of brain metaflammasome proteins

#### PKR-eIF2alpha pathway

**Fig 3:**
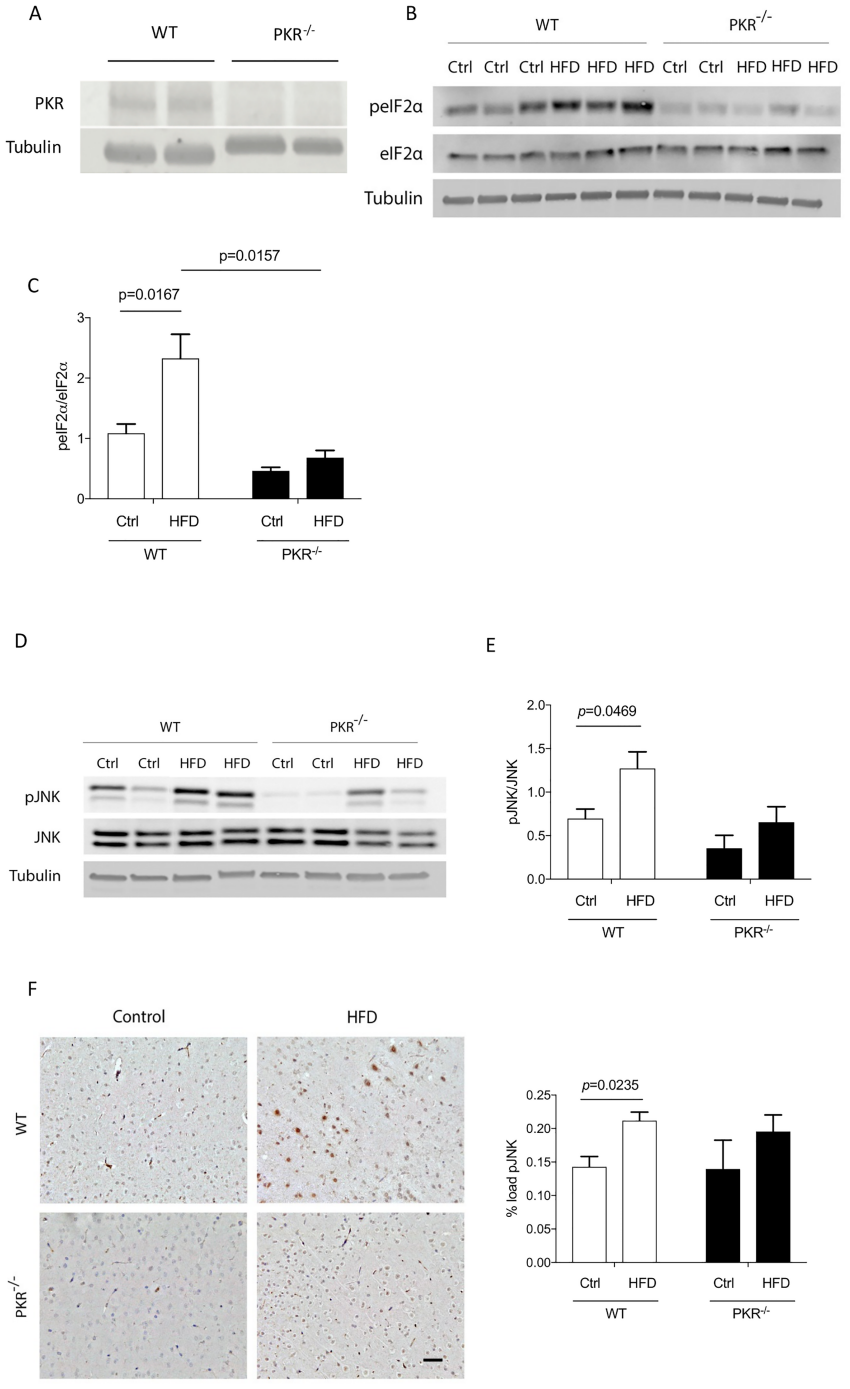
**(A)** Western blot of PKR showing the absence of PKR in the cortex of PKR^-/-^ mice. **(B)** Western blot analysis of peIF2alpha and eIF2alpha in cortex of mice. **(C)** Histogram of the ratio peIF2alpha/eIF2alpha showing a significant increase in the cortex of WT HFD mice (*p* = 0.0167). Error bars = SEM. **(D)** Western blot analysis of pJNK and JNK in cortex of mice. **(E)** Histogram of the ratio pJNK/JNK showing a significant increase in the cortex of WT HFD mice. Error bars = SEM. **(F)** Immunostaining of pJNK in mice cortex and histogram of level of pJNK in the cortex of mice showing a significant increase of pJNK load in the cortex of WT HFD mice. Error bars = SEM. (n = 5).

The results depicted in Fig 3A show the absence of PKR expression in PKR^-/-^ mice. The levels of phosphorylated eIF2alpha (peIF2alpha), a substrate of PKR, was studied by western blot in order to assess the activation of PKR. The results showed a significant increase of peIF2alpha expression levels in the cortex of WT HFD mice (*p* = 0.0167) by 168% compared to WT control mice. In the PKR^-/-^ HFD mice, no significant variation was detected after HFD treatment (*p* = 0.9867). Furthermore, under HFD conditions, the mean expression level of peIF2alpha was significantly lower in the cortex of PKR^-/-^ HFD mice compared to WT HFD mice (*p* = 0.0157) (Fig 3B and 3C).

#### JNK pathway

The results obtained by western blot showed a significant increase of 70% of pJNK expression levels in the cortex of WT HFD mice (*p* = 0.0469). A slight but not significant increase was detected in PKR^-/-^ HFD mice as compared to PKR^-/-^ control mice (*p* = 0.8289). The ratio pJNK/JNK was already lower in PKR ^-/-^ mice before HFD which induced after 16 weeks a non-significant tendency to augment this brain ratio.

Using immunohistochemistry, comparable results were detected in WT mice (*p* = 0.0235) but no significant variation was observed in the cortex of PKR^-/-^ HFD mice compared to PKR^-/-^ control mice (*p* = 0.4239) (Fig 3D, 3E and 3F).

#### pIRS1

**Fig 4:**
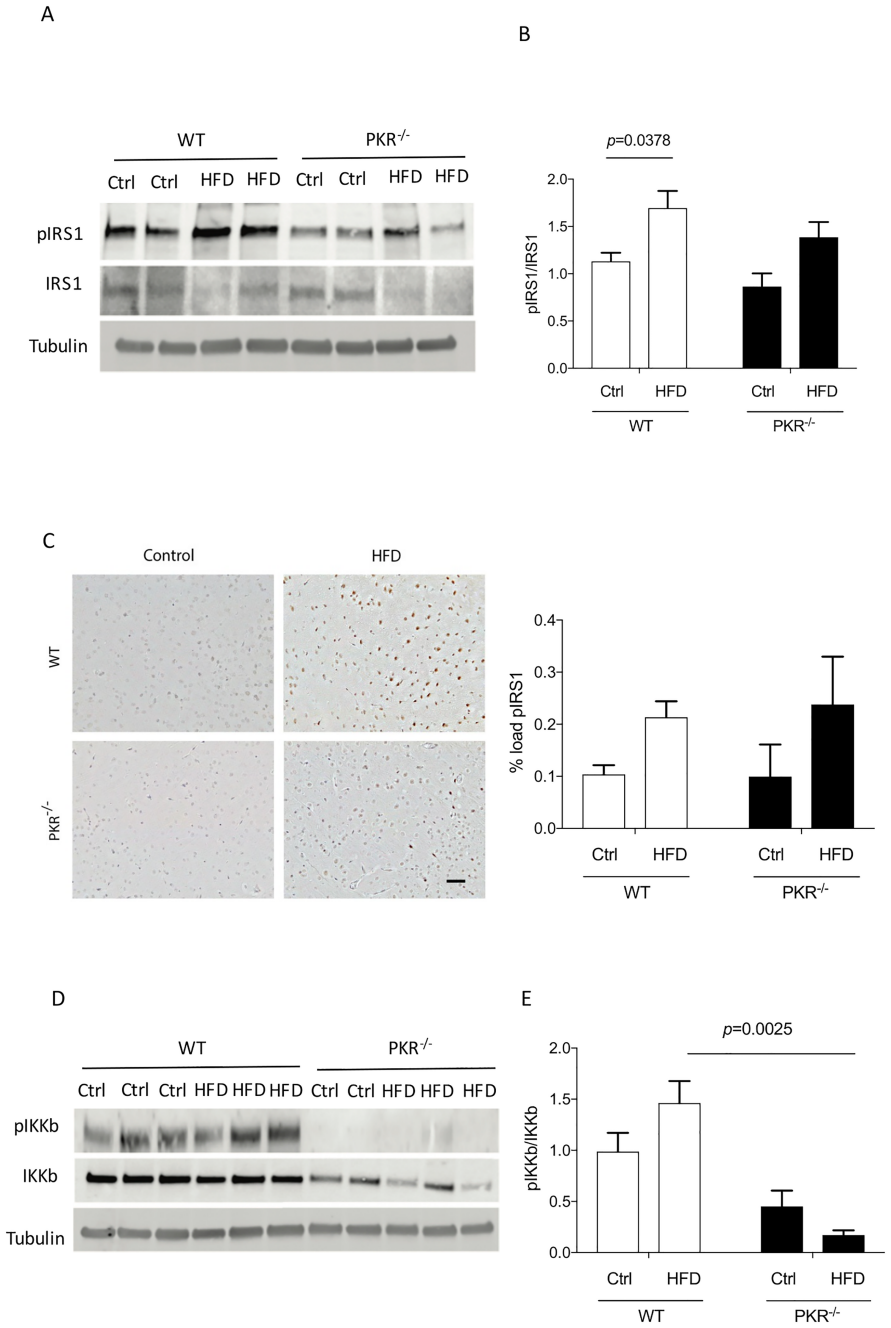
**(A)** Western blot analysis of pIRS1 and IRS1 in cortex of mice. **(B)** Histogram of the ratio pIRS1/IRS1 showing a significant increase in the cortex of WT HFD mice. **(C)** Immunostaining of pIRS in mice cortex and histogram of level of pIRS1 in the cortex of mice did not show any significant change of pIRS1 load in the cortex of both strains. Error bars = SEM. **(D)** Western blot analysis of pIKKbeta and IKKbeta in cortex of mice. **(E)** Histogram of the ratio pIKKbeta/ IKKbeta. A significant change was observed between WT HFD and PKR^-/-^ HFD.

The results using western blot showed a 50% significant increase of pIRS1 expression levels in the cortex of WT HFD mice (*p* = 0.0378). Conversely, no significant difference was detected in the cortex of PKR^-/-^ HFD mice compared to PKR^-/-^ control mice (*p* = 0.0635) The ratio pIRS1/IRS1 was already lower in PKR ^-/-^ mice before HFD which induced after 16 weeks a non-significant tendency to augment this brain ratio. (*p* = 0.4250) (Fig 4A, 4B and 4C).

Our findings are in favor of a reduction of HFD-induced brain insulin resistance in PKR^-/-^ mice. The immunohistochemistry showed non-significant modification of pIRS1 load in the cortex of WT HFD and PKR^-/-^ mice compared to control WT and PKR^-/-^ mice.

#### IKKbeta pathway

The results obtained by western blot showed a trend towards an increase of pIKKbeta levels in the cortex of WT mice exposed to HFD (*p* = 0.267) (Fig 4D and 4E). Also, a trend towards a decrease of pIKKbeta levels was observed in the cortex of PKR^-/-^ HFD mice compared to PKR^-/-^ Ctrl mice (*p* = 0.464). Interestingly, we observed a significant difference of pIKKbeta expression levels between WT HFD mice and PKR^-/-^ HFD mice (*p* = 0.002). These results suggest that the NFκB pathway which is partially modulated by PKR is mildly triggered in the brain of PKR^-/-^ mice.

## Discussion

To our knowledge, no study has explored so far, the presence of a cerebral metaflammasome (PKR, JKN, IKKbeta and IRS1) in the context of experimental obesity and type 2 diabetes. Our results show that the knock-out of PKR in animals with HFD diminishes the abnormal increase of metaflammasome proteins in the brains of experimental animals. In addition, with the exception of insulin levels, no significant difference were noted in terms of body weight and glucose levels between WT mice and PKR^-/-^ mice exposed to HFD. Our results are more in agreement with Lancaster et al results showing that PKR do not control obesity and associated metabolic complications in experimental HFD [5]. Since the role of metaflammasome proteins were highlighted in AD pathophysiology, the modulation of PKR activity could attenuate the detrimental consequences of metabolic disorders on brain signaling pathways even associated with obesity [20]. Diabetes and obesity are co-morbidity factors that increase the risk of AD and this study bring about a valid argument about the role of these pathological conditions on augmented brain PKR activation and consequent abnormal signaling.

In 2010 and 2012, Nakamura et al and Carvalho-Filho et *al*. respectively reported an increased expression of the 4 components of the metaflammasome in peripheral organs (liver, adipose tissue and muscle) of mice after HFD, highlighting the involvement of the metaflammasome in metabolic disorders [3, 4]. In addition, PKR knock-out attenuated most of these peripheral anomalies. These findings were not observed in the study by Lancaster et al [5]. Although PKR does not seem also in this model to attenuate obesity and its metabolic effects, our brain results demonstrates that obesity induces the activation of the brain protein components of the metaflammasome which is partially controlled by PKR. Further studies will be needed to understand the various cerebral and peripheral metabolic abnormalities in HFD treated mice.

### Weight gain, glucose and insulin levels

At the end of HFD period, PKR^-/-^ mice had a mean body weight lower than HFD wild type mice but as seen before the proportional weight gain was comparable in WT and PKR ^-/-^ treated mice. No significant difference was observed in both strains with control diet. This suggests that PKR knock down did not induce by itself a body mass reduction under normal diet conditions. We observed that in WT mice, HFD led to an increase of blood glucose level compared to control mice. This result showed that WT mice exposed to HFD developed type 2 diabetes. Interestingly, in PKR^-/-^ mice, a tendency to increase but no significant difference in blood glucose level was observed between animals fed with HFD and control diet. The oral glucose tolerance test shows no difference between control mice and PKR knockout mice from T0 to T180 minutes but was improved in the report of Carvalho-Filho et *al*. [4]. Overall WT and PKR^-/-^ mice have rather comparable body phenotypes and the only difference was observed under HFD conditions with increased insulin levels in PKR ^-/-^ mice. The reason for the elevated insulin level with moderately increased glucose levels is not known, but could be linked to an enhanced activity of beta cells associated with a degree of peripheral insulin resistance or to a modification of glucagon secretion. These findings could imply that under glucose and lipid stress conditions, the absence of the stress kinase PKR in pancreatic beta cells could lead to a modified control of protein translation leading to increased insulin production. A previous report has shown that increased beta cell function is induced by HFD treatment resulting in increased insulin secretion linked to the activity of tyrosine kinase receptors such as c-KIT [25]. In this report, the authors demonstrated that overexpression of c-KIT in beta cells ameliorates glucose metabolism by increasing insulin secretion and thus implying that this receptor could have a protective role in preventing type 2 diabetes. The question that can be addressed is: how PKR can modulate insulin secretion? A recent study has shown that PKR activated by gluco-lipidotoxicity inhibits cell proliferation in pancreatic beta cells [26]. This finding could indicate that the lack of PKR may prevent an abnormal control of beta cells exposed to HFD. Further studies assessing pancreatic metabolism will be needed to understand the links between glucose and insulin levels in HFD treated PKR ^-/-^ mice.

### Cholesterol level

Our results show in both strains increased cholesterol suggesting that PKR might not have a major role in the total cholesterol level detected during lipid stress. The levels of triglycerides were also studied and showed no variation after HFD treatment for both strains. With the aim to study if HFD induced a dyslipidemia, the levels of HDL and LDL were measured in the plasma of the mice. The results revealed an increase of HDL in both strains after HFD treatment but an increase of LDL was observed just in WT HFD mice. In addition, the ratio LDL/HDL was lower in PKR^-/-^ HFD mice compared to WT HFD mice. These observations suggest that PKR could enhance the production of LDL in response to nutrient excess and that its knock down could alter the production level. Interestingly, the involvement of protein JNK in enhancement of cellular LDL-binding activity has been shown [27]. Knowing that JNK is one of the substrates of PKR, the observations suggest that the knock down of PKR inducing a reduction of activated JNK, could affect the cellular LDL-binding activity and the LDL cholesterol level.

### Microglial cells

Metabolic diseases are associated with a peripheral chronic low-grade inflammation [2] and a link between systemic inflammation and central inflammation has been reported in several studies [28, 29]. We investigated the density of microglia, the brain macrophage, using the marker Iba1 [30] (Fig 2A) and found no difference. A study by Thaler *et al*. in a HFD mouse model reported that astrocytes, can play an important neuroprotective role temporarily limiting neuroinflammation and neuronal loss [28] and neuroprotective mechanism against neuroinflammation is established to limit bystander damages in the brain. Further study assessing brain astrocytic reaction in HFD will be worth trying.

### Metaflammasome proteins

From these findings, we can suggest that peripheral lipid stress can induce the expression of a brain metaflammasome leading to brain insulin resistance through PKR and JNK activation. Furthermore, using PKR^-/-^ mice, we demonstrated that the depletion of PKR could prevent the phosphorylation of the 3 proteins present in the cerebral metaflammasome. Our previous findings with PKR^-/-^ mice have demonstrated that the knockout of the PKR gene 1- prevent cerebral inflammation and amyloid production after LPS systemic administration [31] and 2- is neuroprotective in an experimental model of thiamine deficiency [32]. Although PKR is probably not the only molecular signal at the origin of the brain metabolic anomalies, it may contribute to these abnormal signaling in obese or type 2 diabetes patients together with the toxic accumulation of Abeta in AD brains. Abnormal signaling linked to insulin resistance has been already demonstrated in human AD brain including phosphorylated IRS1 [33] and we have recently shown that metaflammasome proteins are present in AD brains compared to control brains [20]. With our current findings, these previous data argue in favor of a detrimental role for PKR in signaling pathways, which could contribute to add risk factors for AD brain lesions.

## Conclusions

Our results showed that the inhibition of the PKR in animal model could prevent the phosphorylation of JNK, IRS1 and IKKbeta induced in the brain by HFD. However, our results have some limitations. We have used WT mice and not littermate mice due to the lack of possibility of back-crossing PKR^-/-^ mice. We could not determine the exact role of peripheral PKR and brain PKR at the origin of the observed abnormal signaling. There are no brain specific PKR knockout mice and a restoration experiment by a parental permanent supply of PKR in knockout mice could not be done due to the lack of available mouse PKR protein. The other limitation is that the chronic pharmacological inhibition of PKR was not possible because of the toxicity of the current compounds.

Further studies will be needed to determine if the chemical PKR inhibition might represent a valuable new therapeutic target to attenuate brain abnormal metabolic consequences linked to obesity and type 2 diabetes, afford neuroprotection and modulate cognitive decline in affected patients by neurodegenerative diseases such as AD.
